# Correlation analysis between quantitative lung ultrasound parameters and pulmonary function in idiopathic pulmonary fibrosis: an observational study

**DOI:** 10.3389/fmed.2026.1821397

**Published:** 2026-04-23

**Authors:** Ting Zhou, Xing He, Xueming Ju, Chun Zhang, Hui Fang, Yang Liu, Jiao Wang, Chunhong Yang, Lu Guo

**Affiliations:** 1 Department of Pulmonary and Critical Care Medicine, Chengdu Sixth People's Hospital, Chengdu, Sichuan, China; 2 Department of Pulmonary and Critical Care Medicine, Sichuan Provincial People's Hospital, School of Medicine, University of Electronic Science and Technology of China, Chengdu, Sichuan, China; 3 State Key Laboratory of Respiratory Health and Multimorbidity, Department of Pulmonary and Critical Care Medicine, West China Hospital, Sichuan University, Chengdu, Sichuan, China; 4 Department of Ultrasound, Sichuan Academy of Medical Sciences and Sichuan Provincial People's Hospital, School of Medicine, University of Electronic Science and Technology of China, Chengdu, Sichuan, China

**Keywords:** B-lines, disease monitoring, idiopathic pulmonary fibrosis, lung ultrasound, pulmonary function

## Abstract

**Background:**

Idiopathic pulmonary fibrosis (IPF) is a progressive and fatal interstitial lung disease (ILD) with limited monitoring tools. Lung ultrasound (LUS) has emerged as a promising radiation-free alternative, but its clinical utility in IPF requires further validation. This study aimed to comprehensively evaluate the correlation between quantitative LUS parameters and established disease severity markers in IPF patients.

**Methods:**

An observational study was conducted in eligible IPF patients. Participants underwent comprehensive assessment including clinical questionnaires, pulmonary function tests, serum KL-6 measurement, high-resolution computed tomography (HRCT), and a standardized LUS protocol. Correlation and multiple linear regression analyses were performed to examine the relationships between LUS indices and conventional severity markers.

**Results:**

The analysis included 47 IPF patients stratified by DLCO% predicted (≥60% mild, <60% moderate–severe). The moderate–severe group showed significantly higher B-line counts, B-line scores, pleural scores, diaphragm thickness and lower diaphragm thickening fraction (*p <* 0.05). B-line count, B-line score, pleural score, and diaphragmatic thickness all exhibited significant negative correlations with DLCO% predicted (r = −0.84, *p <* 0.01; r = −0.87, *p <* 0.01; r = −0.80, *p <* 0.01; and r = −0.58, *p <* 0.05, respectively). Conversely, the diaphragmatic thickening fraction showed a significant positive correlation with DLCO% predicted (r = 0.44, *p <* 0.05). Multivariable regression analyses identified DLCO% predicted as a significant negative indicator of B-line count (*β* = −0.604, *p <* 0.001), B-line score (*β* = −0.846, *p <* 0.001), and pleural score (*β* = −0.860, *p <* 0.001). SGRQ score was positively associated with both B-line count (*β* = 0.308, *p* = 0.019) and B-line score (*β* = 0.336, *p* = 0.012). Warrick score exhibited a positive association with B-line count (*β* = 0.240, *p* = 0.015) but a negative association with pleural score (*β* = −0.295, *p* = 0.011). BMI also showed a negative association with pleural score (*β* = −0.233, *p* = 0.016).

**Conclusion:**

Quantitative LUS parameters show strong and consistent correlations with clinical, functional, serological, and radiographic markers of IPF severity. These findings support the potential utility of LUS as a comprehensive, non-invasive tool for multidimensional disease assessment in IPF patients.

## Introduction

Idiopathic pulmonary fibrosis (IPF) is a progressive and fatal interstitial lung disease (ILD) with a median survival of 3–5 years after diagnosis (1). Its incidence and prevalence are increasing, particularly in people aged 65 years and older (2). Current diagnostic and monitoring strategies rely heavily on high-resolution computed tomography (HRCT), which, despite its detailed imaging capability, has significant drawbacks including radiation exposure, high cost, limited accessibility, and interobserver variability in interpretation. These limitations underscore the need for a non-invasive, reproducible, and bedside-accessible imaging tool to facilitate auxiliary diagnosis and dynamic monitoring of IPF.

Lung ultrasound (LUS) has gained increasing attention as a point-of-care imaging technique for interstitial lung diseases (3). It is based on the detection of sonographic artifacts arising from altered parenchymal architecture. In interstitial fibrosis, thickened interlobular septa generate hyperechoic vertical artifacts known as B-lines, while fibrosis and subpleural remodeling lead to pleural line irregularities, thickening, and fragmentation. In patients with systemic sclerosis-associated interstitial lung disease, studies have demonstrated that the number of B-lines observed on LUS correlates strongly with the HRCT-based Warrick score, indicating that a higher B-line count corresponds to more severe fibrosis (4, 5). Thus, similar to HRCT, LUS-based B-line quantification can reflect the extent of fibrotic involvement in the lung interstitium. Additional investigations have shown an inverse correlation between the B-line score and functional parameters such as forced vital capacity (FVC) and diffusing capacity for carbon monoxide (DLCO), suggesting that increased B-lines are associated with deteriorated lung function and more pronounced pulmonary impairment (5, 6). Moreover, in patients with connective tissue disease-associated interstitial lung disease (CTD-ILD), the number of B-lines has been closely linked to the intensity of symptoms such as dyspnea, reflecting the underlying fibrosis severity (7). These quantifiable LUS features have therefore demonstrated consistent correlations with both structural and functional deficits in studies of CTD-ILD (8). Compared to HRCT, LUS offers significant advantages: it is radiation-free, low-cost, portable, and allows for real-time dynamic assessment. However, its application has predominantly been investigated in secondary pulmonary fibrosis. Although preliminary studies, such as those by Manolescu et al. (9) have suggested the potential utility of LUS in IPF, these observations have not been rigorously validated through well-designed studies. Consequently, empirical data focusing specifically on idiopathic pulmonary fibrosis populations remain scarce.

This cross-sectional study evaluates the utility of a standardized quantitative LUS protocol (bedside lung ultrasound in emergency protocol) by analyzing correlations between its parameters (B-lines, pleural scores, diaphragmatic function) and established clinical, functional, radiographic, and serological markers of IPF severity.

## Materials and methods

### Study population

**Figure 1:**
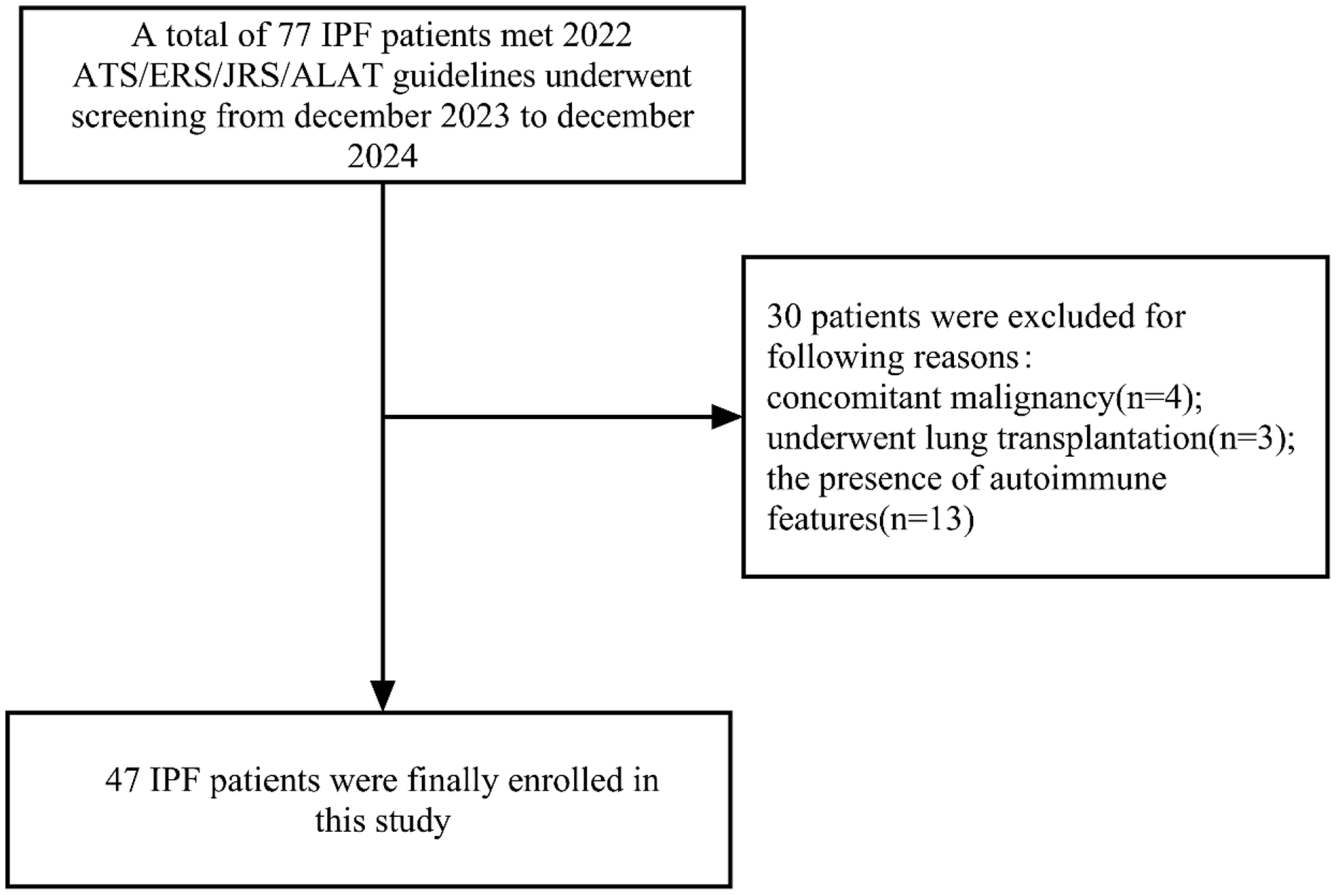
Patient enrollment flowchart.

This observational study enrolled patients diagnosed with idiopathic pulmonary fibrosis (IPF) who met the predefined inclusion criteria. Participants were recruited from the outpatient and inpatient services of the Department of Respiratory Medicine at Sichuan Provincial People’s Hospital, affiliated with the School of Medicine, University of Electronic Science and Technology of China, between December 2023 and December 2024. In the study, patients with IPF were categorized into mild and moderate-to-severe groups based on whether their DLCO% predicted was ≥60%. According to previous studies and clinical guidelines, a DLCO% predicted of 60% is commonly used as a threshold to distinguish mild from moderate-to-severe physiological impairment in interstitial lung disease, and is associated with differences in prognosis and functional status (10). Therefore, patients were stratified accordingly to explore the discriminative capacity of LUS parameters across disease severity stages. The specific screening process is shown in Figure 1. The Ethics Committee of Sichuan Provincial People’s Hospital reviewed and approved the study protocol (reference number: 2023–156).

### Eligibility criteria

*Inclusion criteria*

(1) Diagnosis of IPF is consistent with the 2022 American Thoracic Society/European Respiratory Society/Japanese Respiratory Society/Latin American Thoracic Association (ATS/ERS/JRS/ALAT) clinical practice guidelines (11); (2) Willingness to provide written informed consent and participate in research; (3) Age ≥ 18 years.

*Exclusion criteria*

(1) Established diagnosis of an interstitial lung disease (ILD) other than IPF; (2) Presence of any active or historical malignancy; (3) Concurrent pulmonary infection, pulmonary embolism, significant pleural effusion, acute or chronic heart failure, or acute respiratory distress syndrome; (4) Patients with other neuromuscular diseases affecting respiratory muscle function; (5) History of lung transplantation.

### Data collection

Data collected from patients encompassed demographic and clinical characteristics, including gender, age (years), disease duration (years), body mass index (BMI), smoking history (pack-years), duration of key symptoms (cough and dyspnea), and the presence of comorbidities (e.g., hypertension, diabetes mellitus, and coronary heart disease). Additionally, the following metrics were obtained: dyspnea and health-related quality of life assessments using the St. George’s Respiratory Questionnaire (SGRQ), Borg scale; Functional exercise capacity evaluated via the six-minute walk test (6MWD); pulmonary function tests (PFTs), including spirometry and diffusing capacity for carbon monoxide (DLCO); extent of fibrosis assessed by the HRCT-based Warrick score; serum concentration of Krebs von den Lungen-6 (KL-6); and quantitative lung ultrasound indices such as B-line count, B-line score, pleural line score, diaphragm thickness, diaphragm mobility, and diaphragm thickening ratio.

### HRCT score (12)

All enrolled patients were scanned in the supine position during inspiratory breath-hold using a 64-detector CT scanner (SOMATOM Definition Flash, Siemens, Germany; LightSpeed VCT, GE Medical Systems, USA). The scanning parameters were as follows: 120 kV, window width of 1,000–1,200 HU, window level of −700 to −600 HU, 1-mm slice thickness, 10-mm reconstruction interval, and high-spatial-frequency (bone) algorithm. Subsequent HRCT scans were evaluated by radiologists using the Warrick scoring system. Five abnormalities were assessed: ground-glass opacities, irregular pleural margins, septal/subpleural lines, subpleural cysts, and honeycombing. Each lesion received a severity score (1–5 points, max 15) and an extent score (based on involved segments: 1–3). The alveolitis index (AI, 0–4) was derived from ground-glass scores, while the fibrosis index (FI) summarized scores from the four fibrotic lesions. The total Warrick score (0–30) was the sum of AI and FI, stratified as mild (<8), moderate (8–15), or severe (>15).

### Lung ultrasound evaluation process

Chest and pulmonary ultrasonography was performed utilizing a Samsung HERA W10 color Doppler ultrasound unit equipped with a 3–7 MHz curved abdominal array probe and a 7–12 MHz high-frequency linear transducer. All examinations were conducted with the patient positioned in both the sitting and supine positions by certified sonographers from the Department of Ultrasound at Sichuan Provincial People’s Hospital, LUS examinations were performed by two trained technicians, each with over 3 years of relevant clinical experience. To formally assess interobserver reproducibility prior to the study, both technicians independently examined 10 patients who were not included in the final cohort. Interobserver agreement was evaluated using the intraclass correlation coefficient (ICC) for continuous variables and weighted Cohen’s kappa for categorical scores. The results showed good agreement: ICC for B-line count was 0.72 and weighted kappa for B-line score was 0.73 and for pleural score was 0.70. In cases of inconsistency or significant data discrepancy during the examination, discrepancies were resolved through discussion, taking the average value, or repeating the measurement. The examination adhered to the Bedside Lung Ultrasound in Emergency (BLUE) protocol (13). The specific scanning protocol was as follows: The Upper and Lower BLUE-points were identified by placing the examiner’s hands (approximating the patient’s hand size) flat on the anterior chest wall with the thumbs overlapped. The upper hand was positioned with its little finger immediately inferior to the clavicle and the fingertips aligned with the midline. The little finger of the lower hand indicated the lower anterior pulmonary border, which corresponds to the diaphragmatic line. The combined area covered by both hands approximated the anterior lung field on one side. The Upper BLUE-point was located over the third and fourth metacarpophalangeal joints of the upper hand, while the Lower BLUE-point was identified at the center of the palm of the lower hand. The posterior BLUE-point was designated as the area bounded medially by the paraspinal line and laterally by the scapular line. The Diaphragmatic-point was defined as the intersection of the diaphragmatic line with the midaxillary line. The PLAPS-point (Posterolateral Alveolar and/or Pleural Syndrome point) was determined by the intersection of a horizontal line extending posteriorly from the Lower BLUE-point and the posterior axillary line.

### Ultrasonographic diagnostic criteria

1. Pleural assessment (14)

Pleural Thickness:≤ 0.5 mm: 0 points;0.5 mm to ≤ 1.5 mm: 1 point; >1.5 mm: 2 points. Pleural Surface Morphology: Smooth and regular: 0 points; Continuous but irregular: 1 point; Focal interruption with rough surface: 2 points; Disrupted continuity presenting a “serrated” or “fragmented” pattern: 3 points. Subpleural Nodules: Absent: 0 points; Present: 1 point.

2. B-line Scoring (15, 16)

No or ≤ 2 B-lines per scanned plane: 0 points;3–4 B-lines: 1 point;5–6 B-lines: 2 points; >6 B-lines: 3 points. In cases of B-line confluence where individual counting was not feasible, a semiquantitative assessment was applied based on the percentage of the screen area occupied by coalescent B-lines (e.g., 30% = score 3; 80% = score 8).

### Diaphragmatic excursion and thickening fraction

Diaphragmatic excursion was measured ultrasonographically during quiet breathing. Diaphragmatic thickness was assessed at functional residual capacity (FRC). The diaphragm thickening fraction (DTF) was calculated as follows:


$$\begin{array}{l} \mathrm{DTF}(\%)=\\ \left({\text{Thickness}}_{\mathrm{end}\;\text{deep inspiration}}-{\text{Thickness}}_{\mathrm{end}\;\text{quiet expiration}}\right)\\ /{\text{Thickness}}_{\mathrm{end}\;\text{quiet expiration}}\times 100\% \end{array}$$

### Sample size calculation

A simplified estimation method was used for sample size calculation (17) was used, Based on the recommendation of at least 10 observations per predictor variable for multivariable regression analyses, and anticipating up to 5 predictors in the final models, we aimed for a target sample size of 55 patients after accounting for a 10% potential loss to follow-up. Due to strict inclusion criteria and recruitment challenges during the study period, a final cohort of 47 patients was enrolled.

### Statistical methods

Continuous variables conforming to a normal distribution are presented as mean ± standard deviation (SD), whereas those not conforming to normal distribution are expressed as median with interquartile range (IQR). Categorical variables are summarized as absolute frequencies and/or percentages (%). For comparisons between two groups, independent samples *t*-tests were used for normally distributed continuous variables, and the Mann–Whitney U test was applied for non-normally distributed variables. Pearson or Spearman rank correlation analysis was employed to assess the relationships between quantitative lung ultrasound indices in IPF patients and general demographic characteristics, cardiopulmonary function scores, serum KL-6 levels, HRCT-based Warrick fibrosis scores. Specifically, Pearson correlation analysis was applied for two continuous variables that followed a normal distribution, while Spearman’s test was used for two continuous variables that did not meet the normality assumption, or when one or both variables were categorical. Given the exploratory nature of this study, no adjustment for multiple comparisons (e.g., Bonferroni or FDR) was applied in the correlation analyses; therefore, the findings should be interpreted with caution and considered hypothesis-generating, pending validation in independent cohorts. Multivariable linear regression models were further employed to examine the associations between lung ultrasound parameters (B-line count, B-line score, and pleural score) and clinical, functional, serological, and radiographic markers. In these models, DLCO% predicted was included as a covariate to control for the confounding effect of pulmonary function, allowing for the assessment of independent associations of other variables. All models were constructed using the enter method, with candidate variables selected based on their clinical relevance and statistical significance in correlation analysis. Multicollinearity was assessed using variance inflation factor (VIF) and tolerance, with VIF < 10 and tolerance > 0.1 indicating no significant multicollinearity. The Durbin–Watson test was used to check for independence of residuals, with a value close to 2 suggesting no autocorrelation. All statistical analyses were performed using SPSS version 27.0. A two-sided *p* < 0.05 was considered statistically significant.

## Results

### Patient grouping and baseline characteristics

A total of 77 patients with IPF were screened between December 2023 and December 2024. Of these, 30 were excluded for not meeting the inclusion criteria: 4 had comorbid malignancies, 3 had undergone lung transplantation, and 23 declined participation. Ultimately, 47 patients (41 males and 6 females) were enrolled. Among the 47 enrolled IPF patients, 43 underwent pulmonary function tests (PFTs) for ventilation and diffusion capacity. These patients were divided into two groups based on DLCO% predicted values: those with DLCO% predicted ≥60% were classified as the mild impairment group (mild group), and those with DLCO% predicted <60% were classified as the moderate-to-severe impairment group (moderate-to-severe group). Comparison between the two groups revealed no significant differences in age, gender, duration of cough, disease course, smoking history, pack-year index, smoking cessation status, BMI, prevalence of hypertension, diabetes, coronary heart disease, or diaphragmatic mobility (*p >* 0.05). However, patients in the mild group exhibited a shorter duration of dyspnea and lower SGRQ scores compared to those in the moderate-to-severe group (*p <* 0.05)(Table 1).

**Table 1:** Analysis of differences in patient data stratified by lung function in IPF.

Characteristics	Mild group	Moderate-to-severe group	Z/T/*χ*^2^	*p*
Total number (cases)	23	20		
Male (%)	20 (87.0)	17 (85.0)	1.000	1.000
Age (years)	68.6 ± 7.4	69.7 ± 6.1	−0.525	0.603
BMI (kg/m^2^)	24.3 ± 3.5	22.7 ± 3.5	1.475	0.148
Disease duration (years)	2.8 ± 2.0	3.4 ± 1.8	−0.273	0.787
Cough (years)	2.0 [0.0, 8.0]	3.5 [1.8, 10.8]	−0.860	0.390
Dyspnea (years)	0.5 [0.0, 3.0]	3.0 [1.0, 5.0]	−2.700	0.007^*^
Smoking history (cases)	15 (65.2)	14 (60.9)	0.111	0.739
Smoking index (pack-years)	501.3 ± 339.8	805.7 ± 468.9	−2.012	0.054
Quit smoking (%)	11 (73.3)	13 (92.9)	0.808	0.369
Comorbidities (cases, %)				
Hypertension	11 (47.8)	4 (20.0)	2.524	0.112
Diabetes	5 (21.7)	3 (15.0)	0.030	0.862
Coronary heart disease	1 (4.3)	4 (20.0)	1.255	0.263
SGRQ	14.7 [10.0, 31.0]	30.0 [17.4, 55.1]	−2.459	0.014^*^
Borg Score	1.00 [1.00, 2.00]	2.00 [1.00, 3.75]	−1.636	0.102
6MWD (m)	440.7 ± 89.2	411.1 ± 103.2	1.009	0.319
Serum KL-6 (U/ml)	500.4 [291.0, 657.6]	1009.4 [687.0, 1745.0]	−4.212	<0.001^**^
Warrick score	17.9 ± 4.4	24.6 ± 3.9	−5.185	<0.001^**^
B-line count	13 ± 4	23 ± 4	−9.934	<0.001^**^
B-line score	8 [4, 10]	16 [14, 18]	−5.292	<0.001^**^
Pleural score	17 [14, 25]	34 [30, 36]	−5.023	<0.001^**^
Diaphragm thickness (cm)	0.33 [0.30, 0.42]	0.56 [0.43, 0.67]	−4.247	<0.001^**^
Diaphragm mobility (cm)	3.06 ± 0.56	2.93 ± 0.66	1.128	0.266
Diaphragm thickening ratio (%)	61.27 ± 21.52	48.90 ± 15.15	2.520	0.016^*^

BMI, Body mass index; SGRQ, St. George’s Respiratory Questionnaire; 6MWD, 6-min walk distance; KL-6, Krebs von den Lungen-6; ^*^p < 0.05; ^**^p < 0.001.

### Differences in lung ultrasound findings stratified by lung function

**Figure 2:**
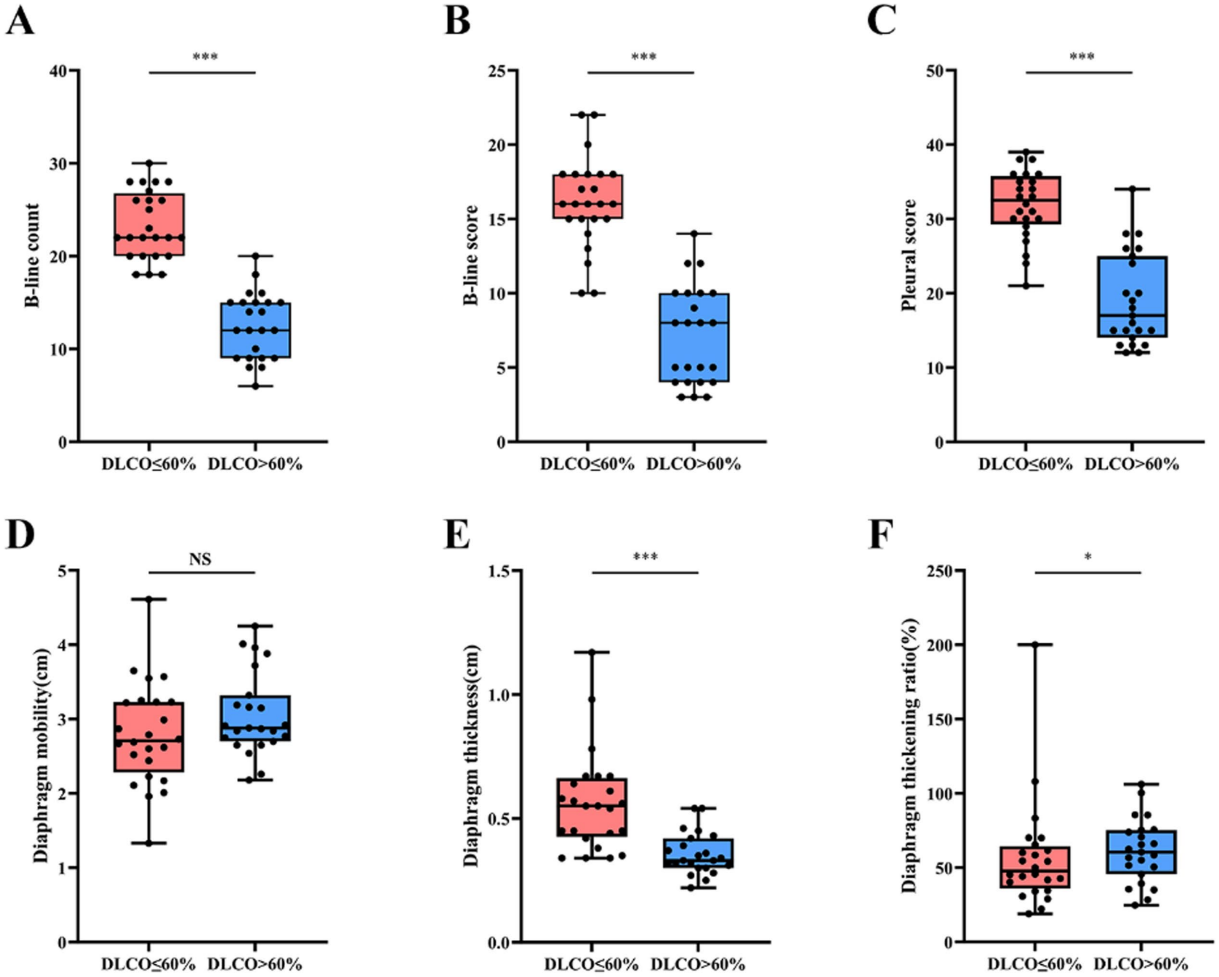
Comparison of lung ultrasound findings in IPF patients by lung function groups. ^***^p < 0.001; ^*^p < 0.05; NS: no statistical significance.

Box plots showing the Analysis of Differences in Lung Ultrasound Parameters Among IPF Patients with Different Lung Function. As illustrated in the figure, patients with IPF and a DLCO% predicted ≤ 60% exhibited significantly higher values in the number of B-lines, B-line score, pleural line score, and diaphragm thickness compared to those with a DLCO% predicted > 60% (*p <* 0.001). Conversely, the diaphragmatic thickening fraction was lower in the former group (Table 1; Figure 2) (*p <* 0.05). No significant difference was observed in diaphragmatic mobility between the two groups (Table 1; Figure 2).

### Correlation analysis of lung ultrasound parameters with clinical indicators

**Figure 3:**
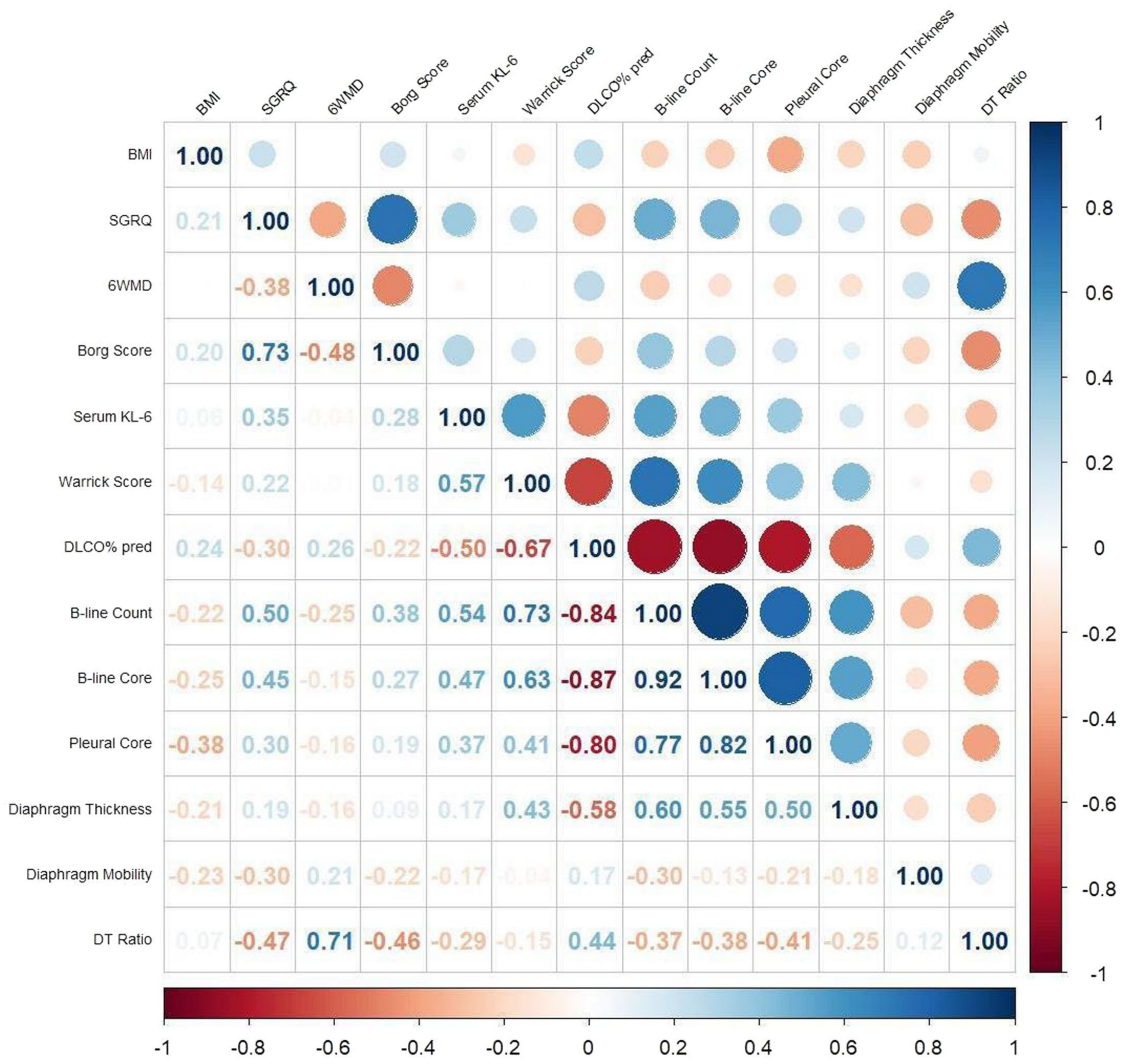
Correlation between quantitative lung ultrasound indicators and BMI, the SGRQ, KL-6, DLCO% predicted, and the Warrick score in patients with idiopathic pulmonary fibrosis.

Correlation analyses revealed that the B-line count showed a significant negative correlation with the 6-min walk distance (6MWD) (r = −0.25, *p <* 0.05) and DLCO% predicted (r = −0.84, *p <* 0.01), and significant positive correlations with the SGRQ (r = 0.50, *p <* 0.01), Borg score (r = 0.38, *p <* 0.01), serum KL-6 level (r = 0.54, *p <* 0.01), and Warrick score (r = 0.73, *p <* 0.01). The B-line score was positively correlated with the SGRQ (r = 0.45, *p <* 0.05), Borg score (r = 0.27, *p <* 0.05), serum KL-6 level (r = 0.47, *p <* 0.05), and Warrick score (r = 0.63, *p <* 0.05), and negatively correlated with DLCO% predicted (r = −0.87, *p <* 0.01). The pleural score was negatively correlated with BMI (r = −0.38, *p <* 0.05), and positively correlated with the B-line count (r = 0.77, *p <* 0.05), B-line score (r = 0.82, *p <* 0.05), SGRQ (r = 0.30, *p <* 0.05), serum KL-6 level (r = 0.37, *p <* 0.05), and Warrick score (r = 0.41, *p <* 0.05). A significant negative correlation was also observed with DLCO% predicted (r = −0.80, *p <* 0.01). Diaphragmatic thickness was positively correlated with the number of B-lines (r = 0.60, *p <* 0.01), B-line score (r = 0.55, *p <* 0.01), pleural line score (r = 0.50, *p <* 0.01), and Warrick score (r = 0.43, *p <* 0.01), and showed a significant negative correlation with DLCO% predicted (r = −0.58, *p <* 0.05) (Figure 3).

Diaphragmatic excursion was negatively correlated with SGRQ (r = −0.30, *p <* 0.05), while the diaphragm thickening fraction was positively correlated with DLCO% predicted (r = 0.44, *p <* 0.05) (Figure 3).

### Multivariable regression analysis of factors influencing LUS parameters

Multiple linear regression analysis was performed to further analyze the factors associated with quantitative lung ultrasound indicators (such as B-line count, B-line score, and pleural line score). The results showed that SGRQ (*β* = 0.308, *p* = 0.019) and Warrick score (*β* = 0.240, *p* = 0.015) positively influenced B-line count. In contrast, DLCO% predicted (*β* = − 0.604, *p <* 0.001) negatively influenced B-line count, and no linear correlation was found between 6MWD, Borg, serum KL-6 level and B-line count (*p* > 0.05) (Table 2). Meanwhile, the results showed that SGRQ (*β* = 0.336, *p* = 0.012) positively influenced B-line score, DLCO% predicted (*β* = −0.846, *p <* 0.001) negatively influenced B-line score, and no linear correlation (*p* > 0.05) was found between Borg score, Warrick score, serum KL-6 level and B-line score (Table 3).

**Table 2:** Multivariable linear regression analysis of the B-line count in IPF patients.

Variables	B (95% CI)	*β*	t	p	F	Adjusted R^2^	Collinearity diagnostics	
							VIF	Tolerance
6WMD	−0.001 (−0.014, 0.011)	−0.017	−0.183	0.856	24.277^**^	0.795	1.840	0.544
SGRQ	0.104 (0.018, 0.189)	0.308	2.470	0.019^*^			2.238	0.447
Borg score	0.343 (−0.553, 1.239)	0.089	0.778	0.442			2.876	0.348
serum KL-6	0.001 (−0.001, 0.003)	0.064	0.729	0.471			1.576	0.635
Warrick score	0.303 (0.063, 0.543)	0.240	2.564	0.015^*^			1.899	0.527
DLCO% predicted	−0.171 (−0.227, −0.115)	−0.604	−6.197	<0.001^**^			1.803	0.555

^*^p < 0.05; ^**^p < 0.01; Durbin-Watson test: 2.119. 6MWD, 6-min walk distance; SGRQ, St. George’s Respiratory Questionnaire; KL-6, Krebs von den Lungen-6; DLCO% predicted, Percentage of predicted diffusing capacity of the lung for carbon monoxide.

**Table 3:** Multivariable linear regression analysis of the B-line score in IPF Patients.

Variables	B (95% CI)	*β*	*t*	*p*	F	Adjusted R^2^	Collinearity diagnostics	
							VIF	Tolerance
SGRQ	0.095 (0.022, 0.167)	0.336	2.659	0.012^*^	22.896^**^	0.785	2.229	0.449
Borg score	−0.455 (−1.216, 0.305)	−0.141	−1.216	0.232			2.193	0.456
Warrick score	−0.003 (−0.228, 0.222)	−0.003	−0.027	0.979			2.031	0.492
Serum KL-6	<0.001 (−0.002, 0.002)	−0.004	0.044	0.965			1.557	0.642
DLCO% predicted	−0.201 (−0.255, −0.146)	−0.846	−7.442	<0.001^**^			1.793	0.558

^*^p < 0.05; ^**^p < 0.01; Durbin-Watson test: 2.130. SGRQ: St. George’s Respiratory Questionnaire; KL-6: Krebs von den Lungen-6; DLCO% predicted: Percentage of predicted diffusing capacity of the lung for carbon monoxide.

Multivariable linear regression analysis of factors associated with the pleural score in IPF patients demonstrated that BMI (*β* = −0.233, *p* = 0.016), Warrick score (*β* = −0.295, *p* = 0.011) and DLCO% predicted (*β* = −0.860, *p <* 0.001) negatively influenced the pleural score and no linear correlation was found between SGRQ, serum KL-6 levels and the pleural score (*p* > 0.05) (Table 4). In the multivariable linear regression models for B-line count, B-line score, and pleural score, collinearity diagnostics confirmed the absence of significant multicollinearity, with all VIF values below 10 and tolerance values above 0.1. Furthermore, the Durbin-Watson test statistics for all models were close to 2, indicating independence of the residuals.

**Table 4:** Multivariable linear regression analysis of the pleural line score in IPF Patients.

Variables	B (95% CI)	*β*	*t*	*p*	F	Adjusted R^2^	Collinearity diagnostics	
							VIF	Tolerance
BMI	−0.557 (−1.003, −0.112)	−0.233	−2.539	0.016^*^	21.243^**^	0.712	1.128	0.886
SGRQ	0.070 (−0.014, 0.154)	0.159	1.684	0.101			1.133	0.882
Warrick score	−0.482 (−0.849, −0.116)	−0.295	−2.668	0.011^*^			2.050	0.488
Serum KL-6	0.001 (−0.002, 0.003)	0.043	0.415	0.681			1.633	0.612
DLCO% predicted	−0.316 (−0.405, −0.228)	−0.860	−7.285	<0.001^**^			1.870	0.535

^*^p < 0.05; ^**^p < 0.01; Durbin-Watson test: 1.802. BMI, body mass index; SGRQ, St. George’s Respiratory Questionnaire; KL-6, Krebs von den Lungen-6; DLCO% predicted, Percentage of predicted diffusing capacity of the lung for carbon monoxide.

## Discussion

IPF is a chronic, progressive pulmonary disease marked by irreversible loss of lung function. This observational study provides a comprehensive evaluation of the relationships between quantitative LUS indices and various clinical parameters, including patient-reported outcomes, physiological measures, serum biomarkers, and HRCT findings in patients with IPF. Our principal findings demonstrate that LUS-derived measures, specifically the B-line count, B-line score, and pleural line score, exhibit significant correlations with disease severity proxies such as the SGRQ score, DLCO% predicted, serum KL-6 levels, and the Warrick score. Furthermore, diaphragmatic parameters showed distinct associations with functional status. Multivariable linear regression analyses confirmed that specific factors, notably the SGRQ, Warrick score, and DLCO% predicted, are independent indicators of certain LUS metrics. These results underscore the potential utility of LUS as a valuable, non-invasive tool for the multidimensional assessment of IPF.

In this study, male patients significantly outnumbered females, and the average age was high, demographic characteristics consistent with previously published global epidemiological features of IPF (18). Group comparisons based on pulmonary impairment revealed that both the B-line count and B-line score were significantly higher in the moderate-to-severe group compared to the mild group. More importantly, correlation analyses revealed strong positive associations between B-line metrics and the SGRQ score, Borg score, serum KL-6 level, and Warrick score, while showing strong negative correlations with DLCO% predicted. These findings are consistent with the pathophysiological understanding of IPF, where progressive fibrotic remodeling and alveolar epithelial injury lead to increased lung density and loss of aeration. B-lines are laser-like vertical artifacts that arise from the reverberation of ultrasound waves at interfaces between aerated and fluid-filled or densified lung tissue. In the context of IPF, the replacement of normal lung architecture with fibrotic tissue, leading to increased lung density and loss of aeration, provides the ideal substrate for the generation of B-lines (19, 20). Therefore, an increase in the number of B-lines and the B-line score is often indicative of a worsening degree of pulmonary fibrosis. The strong positive correlation between B-line count and the Warrick score is particularly significant. The Warrick score, derived from HRCT, is a validated semi-quantitative tool for assessing the extent and severity of fibrotic changes in IPF (18). Our data align with a growing body of recent literature validating LUS against HRCT. A study on B-line ultrasound in systemic sclerosis-associated interstitial lung disease established a significant correlation between the number of B-lines and the Warrick score, and further explored a B-line cut-off value indicative of severe pulmonary fibrosis (21). Similarly, Gargani et al (22) have also established that LUS B-lines correlate with the extent of fibrosis on HRCT, despite differences in assessment methodologies. Furthermore, the significance of B-lines is independent of the specific LUS scanning technique and quantification method used. Additionally, LUS demonstrates high sensitivity in detecting parenchymal alterations, even in the very early stages of ILD, highlighting its potential for monitoring. Our results further substantiate the role of LUS as a viable, radiation-free alternative for serial assessment of fibrotic burden. Pleural scores correlated negatively with BMI, to the best of our knowledge, this finding has not been described in previous comparable studies and its accuracy requires further validation. We hypothesize that this result may be due to the masking of subpleural lesions by adipose tissue, and positively with SGRQ, the Warrick score and KL-6, linking pleural abnormalities to symptomatic and biological disease activity. More intriguing is the sign reversal of the Warrick score in the multivariable model, where it showed a negative association with the pleural score despite a positive univariable correlation. This likely reflects that DLCO% predicted acts as a strong mediator, suggesting that after accounting for functional impairment, the Warrick score and the pleural score may reflect different dimensions of disease pathology. Diaphragmatic thickness is a key indicator for assessing diaphragmatic atrophy, with normal thickness above 2 mm; values below 2 mm suggest possible atrophy. The diaphragmatic thickening fraction reflects the contractile performance of the diaphragm itself, in normal adults during quiet breathing, it typically ranges between 20 and 36% (23, 24). Diaphragmatic parameters were assessed via right-sided ultrasound to avoid interference from gastric and intestinal air (25). The mild impairment group showed a higher diaphragmatic thickening fraction, indicating compensatory adaptation, and supporting diaphragmatic function as a marker of disease severity. No significant difference was observed in diaphragmatic excursion, likely because it more closely reflects ventilatory rather than diffusive capacity. Multivariable regression confirmed independent associations between B-line metrics, pleural scores, SGRQ, and DLCO% predicted, supporting their prognostic relevance.

Although this study yielded certain results, several limitations should be acknowledged. First, this was a single-center study with a small sample size, limiting the generalizability and accuracy of the findings. Specifically, the events-per-variable ratio in our multivariable regression models was approximately 8–9, which is below the commonly recommended threshold of 10–20. This raises the possibility of model overfitting and may affect the stability and generalizability of the regression coefficients. Therefore, our findings should be considered exploratory and hypothesis-generating, warranting validation in larger, independent cohorts. Additionally, the cross-sectional design of this study precludes conclusions about longitudinal monitoring utility, and the potential for LUS to serve as a monitoring tool requires prospective validation. Furthermore, ROC analysis to determine diagnostic thresholds was not performed, as this requires larger cohorts beyond the scope of this exploratory study. Future diagnostic accuracy studies are needed to evaluate the clinical utility of LUS in IPF. Second, selection bias may exist as the study only included surviving patients who had not undergone lung transplantation, while those with severe disease or recovery were not represented. Third, although multivariable linear regression was used to adjust for known confounding factors, unmeasured or unknown confounders may still affect the results, leading to biased associations. Additionally, factors such as comorbidities, specific treatment regimens such as antifibrotic therapy, corticosteroids, oxygen therapy, and pulmonary rehabilitation, and acute exacerbations can influence the prognosis of IPF patients but were not systematically recorded or adjusted for in this study. Future studies could employ stratified analyses or incorporate randomized controlled trial designs to enhance the reliability of the results. Fourth, the study lacked histopathological and genetic analyses, limiting insight into the molecular basis of IPF progression. Fifth, the BLUE protocol has rarely been applied in IPF outpatients or inpatients, so data on its use for chronic disease monitoring remain limited. Sixth, lung ultrasound measurements are susceptible to human factors such as operator experience and patient respiratory status, introducing subjectivity. Additionally, while interobserver reliability was assessed, intraobserver reliability and Bland–Altman analysis were not performed. Furthermore, multiple correlation tests were performed without adjustment for multiple comparisons, which may increase the risk of type I error. Therefore, these findings should be interpreted as exploratory and require validation in future studies. Future multicenter studies with larger sample sizes, extended follow-up, and improved control of confounding factors are needed to validate these findings.

## Conclusion

This study used the BLUE protocol to quantify lung lesions and diaphragmatic function in IPF patients. LUS parameters (B-line count, B-line score and the pleural line score) were correlated with clinical indicators, dyspnea scores, quality of life, 6MWD, pulmonary function, HRCT scores, and serum KL-6 levels. These associations provide a foundation for future development of an integrated risk prediction and chronic disease management model. Future multicenter randomized controlled trials with larger cohorts and longer follow-up are warranted. Standardization of LUS protocols, along with integration of multimodal imaging, biomarkers, genomics, and functional assessments, will be essential to elucidate the heterogeneous progression of IPF and facilitate early diagnosis and personalized management.

## Data Availability

The raw data supporting the conclusions of this article will be made available by the authors, without undue reservation.
